# Time interval from diagnosis to treatment of brain metastases with stereotactic radiosurgery is not associated with radionecrosis or local failure

**DOI:** 10.3389/fonc.2023.1132777

**Published:** 2023-03-20

**Authors:** Justin Leu, Meredith Akerman, Christopher Mendez, Jonathan W. Lischalk, Todd Carpenter, David Ebling, Jonathan A. Haas, Matthew Witten, Marissa Barbaro, Paul Duic, Lee Tessler, Michael C. Repka

**Affiliations:** ^1^ Renaissance School of Medicine, Stony Brook University, Stony Brook, NY, United States; ^2^ Division of Health Services Research, New York University (NYU) Long Island School of Medicine, Mineola, NY, United States; ^3^ Department of Radiation Oncology, Perlmutter Cancer Center at New York University (NYU) Long Island, Mineola, NY, United States; ^4^ NYCyberKnife at Perlmutter Cancer Center – Manhattan, New York, NY, United States; ^5^ Department of Medical Physics, Perlmutter Cancer Center at New York University (NYU) Long Island, Mineola, NY, United States; ^6^ Department of Neurology, New York University (NYU) Long Island School of Medicine, Mineola, NY, United States; ^7^ Department of Neurosurgery, Perlmutter Cancer Center at New York University (NYU) Long Island, Mineola, NY, United States; ^8^ Department of Radiation Oncology, University of North Carolina School of Medicine, Chapel Hill, NC, United States

**Keywords:** stereotactic radiosurgery, brain metastases, immunotherapy, radionecrosis, treatment delays, cancer, radiation therapy, radiation necrosis

## Abstract

**Introduction:**

Brain metastases are the most common intracranial tumor diagnosed in adults. In patients treated with stereotactic radiosurgery, the incidence of post-treatment radionecrosis appears to be rising, which has been attributed to improved patient survival as well as novel systemic treatments. The impacts of concomitant immunotherapy and the interval between diagnosis and treatment on patient outcomes are unclear.

**Methods:**

This single institution, retrospective study consisted of patients who received single or multi-fraction stereotactic radiosurgery for intact brain metastases. Exclusion criteria included neurosurgical resection prior to treatment and treatment of non-malignant histologies or primary central nervous system malignancies. A univariate screen was implemented to determine which factors were associated with radionecrosis. The chi-square test or Fisher’s exact test was used to compare the two groups for categorical variables, and the two-sample t-test or Mann-Whitney test was used for continuous data. Those factors that appeared to be associated with radionecrosis on univariate analyses were included in a multivariable model. Univariable and multivariable Cox proportional hazards models were used to assess potential predictors of time to local failure and time to regional failure.

**Results:**

A total of 107 evaluable patients with a total of 256 individual brain metastases were identified. The majority of metastases were non-small cell lung cancer (58.98%), followed by breast cancer (16.02%). Multivariable analyses demonstrated increased risk of radionecrosis with increasing MRI maximum axial dimension (OR 1.10, p=0.0123) and a history of previous whole brain radiation therapy (OR 3.48, p=0.0243). Receipt of stereotactic radiosurgery with concurrent immunotherapy was associated with a decreased risk of local failure (HR 0.31, p=0.0159). Time interval between diagnostic MRI and first treatment, time interval between CT simulation and first treatment, and concurrent immunotherapy had no impact on incidence of radionecrosis or regional failure.

**Discussion:**

An optimal time interval between diagnosis and treatment for intact brain metastases that minimizes radionecrosis and maximizes local and regional control could not be identified. Concurrent immunotherapy does not appear to increase the risk of radionecrosis and may improve local control. These data further support the safety and synergistic efficacy of stereotactic radiosurgery with concurrent immunotherapy.

## Introduction

Brain metastases are the most common intracranial tumor diagnosed in adults with an incidence that far outpaces that of primary malignant brain tumors (1). The incidence rate of brain metastases is approximately 9%-17%, and rates appear to be increasing particularly for patients with breast cancer, colorectal cancer, lung cancer, and melanoma due to improvements in systemic therapies, cancer surveillance, and overall patient survival (2, 3). Historical management of brain metastases with radiotherapy consisted of whole brain radiation therapy (WBRT), which delivers a uniform, low dose of radiation to the entire brain but is also associated with cognitive decline, fatigue, and alopecia among other symptoms (4). The development of stereotactic radiosurgery (SRS) in 1961 allowed a combination of precise localization with a steep dose gradient to treat brain metastases with a much higher biologically effective dose (BED) while sparing the uninvolved brain from a substantial radiation dose, altering the paradigm of brain radiotherapy (5, 6).

Today, SRS is utilized as a monotherapy as well as in conjunction with both WBRT and surgical resection (7, 8). Despite the higher rates of distant intracranial failure associated with SRS, local control is similar or better than with WBRT and salvage SRS can be offered if new intracranial metastases develop after treatment (9, 10). Unfortunately, SRS carries an increased risk of radionecrosis, with an incidence of approximately 20-30%, compared to the negligible incidence in patients treated with WBRT (11–13). Radionecrosis is a delayed toxicity of radiotherapy which can occur months to years following administration of SRS (14). While the precise pathophysiology of radionecrosis remains imperfectly characterized, the process is likely mediated through a combination of vascular insult, glial cell damage, and aggressive inflammatory response (15, 16). Furthermore, definitive diagnosis can be elusive due to similarities in appearance between radionecrosis and recurrent tumor (17, 18).

To date, several patient-related risk factors for radionecrosis have been identified. Evidence suggests that different areas of the brain may be more radiosensitive and therefore more prone to radionecrosis than others. Extrapolating from treatment of arteriovenous malformations (AVMs), the brainstem appears to be more resistant to the development of radionecrosis, while the frontal cortex may be more radiosensitive (19). Furthermore, radiosurgical treatment of peripheral metastases has also been associated with lower rates of radionecrosis, likely secondary to radiation “dose-dumping” into the non-neuronal tissues such as the calvarium (20). Limited data may suggest that certain tumor histologies are more susceptible to radionecrosis than others (21). In addition, increasing tumor size was identified early as a negative prognostic factor for development of radionecrosis, and consequently has been included as a stratification factor in every landmark randomized trial on the topic (22–25).

Treatment-related risk factors have also been identified including radiosurgical dose as a well-established predictor of radionecrosis (26–28). Rather than delivering radiation in a single dose, fractionated SRS may lower rates of radionecrosis when treating lesions over 2 cm, according to retrospective data, especially when the volume of normal brain receiving less than 18 Gy can be limited to 30 cc or less (29–31). Given the use of SRS in combination with WBRT and as a salvage treatment in the setting of recurrent disease, a history of prior radiation treatment and the time interval between treatments can also influence the incidence of radionecrosis (32, 33). Regarding concurrent immunotherapies, particularly with the increases in indications for immunotherapies and the long half-lives of many new therapeutic agents, multiple observational studies have shown that concurrent immunotherapies may improve local and regional disease control but also exacerbate the risk of radionecrosis (34–44).

However, it is not known whether increasing the time interval between diagnosis of brain metastases and radiosurgery is associated with changes in the incidence of radionecrosis or regional disease control and there are limited data available on changes in the incidence of local disease control (45). As asymptomatic brain metastases are not considered an oncologic emergency, there is no standardized time frame between diagnosis and radiation treatment. Furthermore, this time frame may vary by patient preference, insurance authorization time, receipt of recent chemotherapy, and availability of the radiation oncologist, neurosurgeon, dosimetrist, and radiation physicist. Brain metastases may significantly increase in size within days to weeks with average growth rates ranging from approximately 0.012 to 0.040 cm^3^ per day depending on histology (46–48). In this report, we seek to identify an optimal time interval between diagnosis of brain metastases and treatment that maximizes local and regional control while minimizing the incidence of radionecrosis.

## Materials and methods

### Patient eligibility

This single institution, retrospective study was approved by the Institutional Review Board (S20-01539). The patient population consisted of all patients who received stereotactic radiosurgery (single fraction) or fractionated stereotactic radiosurgery (between two and five fractions) for intact brain metastases at NYU Langone Long Island Hospital from 8/22/2011 to 8/16/2021 using a frameless, robotic radiosurgery technique with the CyberKnife^®^ (Accuray Inc., Sunnyvale, CA, USA) platform. Exclusion criteria included lack of follow-up imaging, neurosurgical resection prior to treatment with subsequent post-operative radiosurgery, treatment of non-malignant intracranial targets (e.g. AVM, trigeminal neuralgia, meningioma, vestibular schwannoma, pituitary adenoma), or treatment of primary central nervous system malignancy. A history of previous WBRT was allowed. A total of 220 patients were screened, 45 patients were excluded due to lack of follow-up imagining, and 68 patients were excluded due to other exclusion criteria.

### Methods and procedures

In general, patients underwent clinical evaluation and surveillance MRI of the brain at least every 3-6 months following treatment until local failure, regional intracranial progression, or death. Clinical and therapeutic data were abstracted from multiple medical records: ARIA (Varian Medical Systems, Palo Alto, CA, USA), Precision (Accuray Inc., Sunnyvale, CA, USA), and EPIC (Epic Systems Corporation, Verona, WI, USA). Late toxicity was scored according to the National Cancer Institute Common Terminology Criteria for Adverse Events, Version 5.0 (NCI-CTCAE 5.0). In order to determine whether delays in treatment were associated with clinical outcomes, time interval between diagnostic MRI and treatment, as well as time interval between CT simulation and treatment were recorded for all patients. Gross tumor volume (GTV) and planning target volume (PTV) were reported as volumetric measures. Local progression was scored according to the Response Assessment in Neuro-Oncology (RANO) criteria for brain metastases. Immunotherapy (ITX) was defined as concurrent if it was delivered within 14 days of radiosurgery (34). Regional failure, or distant brain failure (DBF), was defined as the development of one or more new brain metastases at a distant untreated site. Radionecrosis was determined by review of the relevant imaging and radiology report, as well as clinical documentation by the radiation oncologist, neurosurgeon, and neuro-oncologist, unless histopathologic confirmation was available.

### Statistical analysis

A univariate screen was implemented to determine which factors were associated with radionecrosis. Continuous data are reported as mean ± standard deviation or median (25th, 75th percentiles), while categorical data are reported as frequency and percent. The chi-square test or Fisher’s exact test, as deemed appropriate, was used to compare the two groups for categorical variables, and the two-sample t-test or Mann-Whitney test was used for continuous data. Those factors that appeared to be associated with radionecrosis on univariate analyses (using a pre-specified p-value of <0.10) were included in a multivariable model. Multicollinearity was checked using the variance inflation factor (VIF), which assesses how much the variance of an estimated regression coefficient increases if the predictors are correlated. A cutoff of VIF > 10 was used to remove variables that were correlated with one another. Generalized Estimating Equations (GEE) (49, 50) were used as a method of parameter estimation for the correlated binary data of radionecrosis (clustered within a subject), with an exchangeable correlation matrix (PROC GENMOD). Analyses were performed on a per-lesion basis.

Time to local failure and time to regional failure were analyzed using standard methods of survival analysis. In cases where the endpoint event, “local failure” or “regional failure,” had not yet occurred, the number of months until last follow-up was used and considered ‘censored.’ Kaplan-Meier product limit curves were constructed, where the data were stratified by immunotherapy. The groups were compared using the log-rank test. Univariable and multivariable Cox proportional hazards models were used to assess potential predictors of time to local failure and time to regional failure. Results are reported as hazard ratios with corresponding 95% confidence intervals (PROC PHREG).

Unless otherwise specified, a result was considered statistically significant at the p=0.05 level of significance. All analyses were performed using SAS version 9.4 (SAS Institute Inc., Cary, NC, USA).

## Results

### Patient cohort characteristics

A total of 107 evaluable patients were identified with a total of 256 individual brain metastases (**Table 1**). The median number of metastases per patient was 2 (1, 3). The median follow-up time was 444 (282, 771) days, with a median follow-up in living patients of 502 (307.5, 816) days. The mean age of this patient cohort was 65.43 years with a standard deviation of 9.70. There were 42 males (39.25%) and 65 females (60.75%). The majority of metastases were non-small cell lung cancer (NSCLC) histology (n=151, 58.98%), followed by breast cancer (n=41, 16.02%), small cell lung cancer (SCLC) (n=14, 5.47%), renal cell carcinoma (RCC) (n=13, 5.08%), melanoma (n=11, 4.3%), prostate (n=1, 0.39%), and other cancers (n=25, 9.77%). The median maximum axial dimension of the brain metastases in millimeters was 7 (4, 12). The median time period between initial diagnostic MRI and first treatment was 34 (28, 48.5) days, and the median time period between CT simulation and first treatment was 14 (11, 18.5) days.

**Table 1:** Univariate analyses comparing radionecrosis (Yes vs. No).

Parameter	Total brain metastases (n=256)	Radionecrosis		*p*-value
		No (n=199)	Yes (n=57)	
**Age at Diagnosis**	65.43 ± 9.70	66.04 ± 9.68	63.33 ± 9.58	0.0637
Gender (Male)*	42 (39.25%)	31 (38.75%)	11 (40.74%)	0.8547
**Histology**				0.8790
*** Breast***	41 (16.02%)	33 (16.58%)	8 (14.04%)	
*** NSCLC***	151 (58.98%)	116 (58.29%)	35 (61.4%)	
*** Other***	64 (25.00%)	50 (25.13%)	14 (24.56%)	
**MRI Max Axial Dimension (mm)**	7 (4, 12)	7 (4, 11)	10 (6, 16)	0.0021
**Dx MRI - First Tx Interval**	34 (28, 48.5)	34 (29, 49)	34 (26, 48)	0.5997
**Sim First Tx Interval**	14 (11, 18.5)	14 (11, 19)	14 (10, 17)	0.9214
**Fractions**				0.5821
*** 1***	217 (84.77%)	170 (85.43%)	47 (82.46%)	
*** >1***	39 (15.23%)	29 (14.57%)	10 (17.54%)	
**Total Dose (cGy)**	2000 (2000, 2000)	2000 (2000, 2000)	2000 (1800, 2000)	0.7736
**Rx IDL (%)**	80.8 (79, 84)	80.8 (78.5, 83)	82 (79.2, 84.8)	0.0491
**GTV (cc)**	0.42 (0.17, 1.33)	0.32 (0.14, 1.23)	0.68 (0.36, 2.08)	0.0025
**PTV (cc)**	0.98 (0.45, 2.55)	0.85 (0.38, 2.31)	1.55 (0.73, 3.47)	0.0077
**Max GTV Dose (cGy)**	2427 (2273, 2561)	2427 (2278, 2547)	2410 (2194, 2564)	0.7102
**Normal Brain Constraint**	80 (31.25%)	60 (30.15%)	20 (35.09%)	0.4783
**Concurrent ITX**	106 (41.41%)	86 (43.22%)	20 (35.09%)	0.3443
**Previous SRS other lesion**	75 (29.30%)	59 (29.65%)	16 (28.07%)	0.8175
**Previous SRS same lesion**	0 (0%)	0 (0%)	0 (0%)	*N/A*
**Previous WBRT**	26 (10.16%)	13 (6.53%)	13 (22.81%)	0.0003

Age was reported as mean ± standard deviation, remaining continuous data was reported as median (25th, 75th percentiles), and categorical data was presented as frequency (percent).

* Based on the 107 total subjects.

### Treatment information

The majority of metastases (n=217, 84.77%) were treated with single fraction SRS while the remainder (n=39, 15.23%) were treated with fractionated SRS (**Table 1**). For patients treated with single fraction SRS, the median total dose was 2000 cGy (1800 cGy, 2000 cGy), the median prescription isodose line (Rx IDL) was 80.80% (78.70%, 84%), the median GTV was 0.35 mL (0.16 mL, 1.04 mL), the median PTV was 0.85 mL (0.42 mL, 1.83 mL), and the median maximum GTV dose was 2404 cGy (2238 cGy, 2500 cGy). For patients treated with fractionated SRS, the median total dose was 2500 cGy (2250 cGy, 2700 cGy), the median Rx IDL was 80.80% (79%, 82.50%), the median GTV was 2.45 mL (0.31 mL, 12.32 mL), the median PTV was 3.78 mL (0.98 mL, 15.12 mL), and the median maximum GTV dose was 3165 cGy (2768 cGy, 3333 cGy). A minority of metastases (n=80, 31.25%) were treated with a normal brain constraint. Seventy-six (35.02%) metastases treated with single fraction SRS utilized a normal brain constraint, while 4 (10.26%) metastases treated with fractionated SRS utilized a normal brain constraint. Nearly half of metastases were also treated with concurrent ITX (n=106, 41.41%). Seventy-five (29.30%) metastases had a history of previous SRS treatment for different metastases in the same patient, while there were no metastases identified that were previously treated with SRS. A small percentage (n=26, 10.16%) of metastases had a history of prior WBRT.

### Association between radionecrosis and other variables

The overall incidence of radionecrosis in this patient cohort was 22.27% (n=57). Of the metastases with radionecrosis, 24 (42.11%) were asymptomatic, 25 (43.86%) presented with moderate symptoms and were treated with corticosteroids or bevacizumab, and 8 (14.04%) metastases presented with severe symptoms requiring medical intervention such as surgery. Nine (15.79%) instances of radionecrosis were diagnosed using histopathology, and 48 (84.21%) instances of radionecrosis were diagnosed after review of radiographic imaging in consultation with the treating physicians. The median time to development of radionecrosis was 221 (103, 378) days. After univariate analyses, gender, tumor histology, time interval between diagnostic MRI and first treatment, time interval between CT simulation and first treatment, fractionated versus single fraction SRS, total dose, max GTV dose, the presence of a normal brain constraint, concurrent ITX, and previous SRS to another lesion were not associated with radionecrosis (**Table 1**). Conversely, the maximum axial dimension on MRI (p=0.0021), Rx IDL (p=0.0491), GTV (p=0.0025), PTV (p=0.0077), and a history of previous WBRT (p=0.0003) were all associated with radionecrosis. A trend was observed for the association between age at diagnosis (p=0.0637) and radionecrosis, so age at diagnosis was consequently included in the multivariable analyses for radionecrosis (**Table 2**). Unsurprisingly, multicollinearity analysis demonstrated high correlation between GTV and PTV; PTV was excluded from multivariable analyses. After multivariable analyses, only MRI maximum axial dimension (OR 1.10, 95% CI 1.02 – 1.19, p=0.0123) and a history of previous WBRT (OR 3.48, 95% CI 1.18 – 10.28, p=0.0243) were associated with an increased risk of radionecrosis.

**Table 2:** Multivariate Generalized Estimating Equations (GEE) for radionecrosis.

Parameter		Beta Estimate	Standard Error	Odds Ratio	95% Confidence Intervals for the Odds Ratio		*p*-value
**Intercept**		-6.70	4.98				0.1785
**Age at Diagnosis**		-0.04	0.02	0.96	0.93	1.00	0.0658
**MRI Max Axial Dimension (mm)**		0.10	0.04	1.10	1.02	1.19	0.0123
**Rx IDL**		0.09	0.06	1.09	0.97	1.22	0.1406
**GTV (cc)**		-0.08	0.05	0.92	0.83	1.02	0.0987
**Previous WBRT**	*Yes*	1.25	0.55	3.48	1.18	10.28	0.0243
	*No*	*ref*					

### Association between local failure and other variables

**Figure 1:**
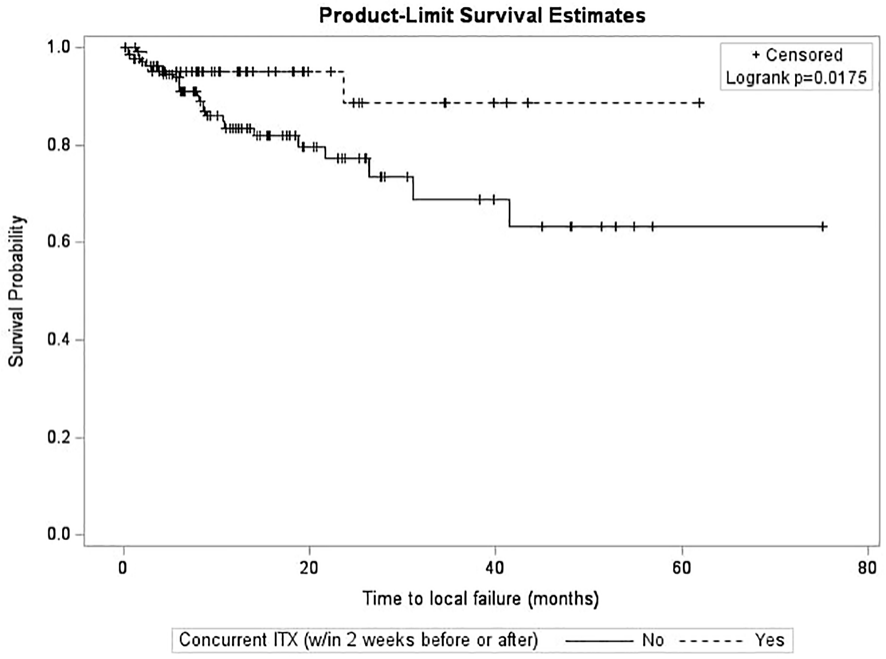
Kaplan-Meier curves for time to local failure stratified by immunotherapy.

After survival analysis and construction of Kaplan-Meier product limit curves, metastases treated with concurrent ITX demonstrated significantly better local failure-free survival (log-rank p=0.0175) compared to metastases treated without concurrent ITX (**Figure 1**). On univariate analyses, age at diagnosis, time interval between diagnostic MRI and first treatment, time interval between CT simulation and first treatment, fractionated versus single fraction SRS, total dose, max GTV dose, presence of normal brain constraint, previous SRS for a different lesion, and a history of WBRT were not associated with local failure (**Table 3**). NSCLC histology compared to other histologies (HR 0.31, 95% CI 0.14 – 0.69, p=0.0040), Rx IDL (HR 0.90, 95% CI 0.82 – 0.99, p=0.0256), and concurrent ITX (HR 0.35, 0.14 – 0.87, p=0.0232) were associated with a decreased risk of local failure. Conversely, increasing MRI maximum axial dimension (HR 1.05, 95% CI 1.01 – 1.10, p=0.0078), GTV (HR 1.07, 95% CI 1.04 – 1.10, p=0.0001), and PTV (HR 1.06, 95% CI 1.04 – 1.08, p=0.0001) were associated with an increased risk of local failure. Furthermore, a trend was observed for an association between male gender (HR 1.99, 95% CI 0.99 – 3.99, p=0.0532) and increased risk of local failure. Again, secondary to substantial collinearity, PTV was excluded from the multivariable analyses. After multivariable analyses, NSCLC histology (HR 0.23, 95% CI 0.09 – 0.58, p=0.0018) and concurrent ITX (HR 0.31, 95% CI 0.12 – 0.81, p=0.0159) were associated with a lower risk of local failure, while male gender (HR 3.73, 95% CI 1.46 – 9.52, p=0.0059) and increasing GTV (HR 1.09, 95% CI 1.06 – 1.13, p=0.0001) were associated with an increased risk of local failure.

**Table 3:** Univariable and multivariable analyses for time to local failure.

Parameter		UNIVARIABLE				MULTIVARIABLE			
		Hazard ratio	95% Confidence Limits for the Hazard Ratio		*p*-value	Hazard ratio	95% Confidence Limits for the Hazard Ratio		*p*-value
**Age at Diagnosis**		0.98	0.95	1.02	0.4174				
**Histology**	*Breast*	0.64	0.25	1.62	0.3481	1.63	0.52	5.10	0.4016
	*NSCLC*	0.31	0.14	0.69	0.0040	0.23	0.09	0.58	0.0018
	*Other*	*ref*				*ref*			
**Gender**	*Male vs. Female*	1.99	0.99	3.99	0.0532	3.73	1.46	9.52	0.0059
**MRI Max Axial Dimension (mm)**		1.05	1.01	1.10	0.0078				
**Dx MRI - First Tx Interval**		1.00	0.99	1.01	0.6804				
**Sim First Tx Interval**		1.03	0.98	1.08	0.2251				
**Fractions**	*>1 vs. 1*	1.36	0.47	3.93	0.5751				
**Total Dose (cGy)**		1.00	1.00	1.00	0.7641				
**Rx IDL (%)**		0.90	0.82	0.99	0.0256				
**GTV (cc)**		1.07	1.04	1.10	<0.0001	1.09	1.06	1.13	<0.0001
**PTV (cc)**		1.06	1.04	1.08	<0.0001				
**Max GTV Dose (cGy)**		1.00	1.00	1.00	0.0657				
**Normal Brain Constraint**	*Yes vs. No*	1.32	0.64	2.75	0.4546				
**Concurrent ITX**	*Yes vs. No*	0.35	0.14	0.87	0.0232	0.31	0.12	0.81	0.0159
**Previous SRS other lesion**	*Yes vs. No*	1.44	0.68	3.06	0.3421				
**Previous WBRT**	*Yes vs. No*	0.48	0.11	2.01	0.3137				

### Association between regional failure (distant brain failure) and other variables

**Figure 2:**
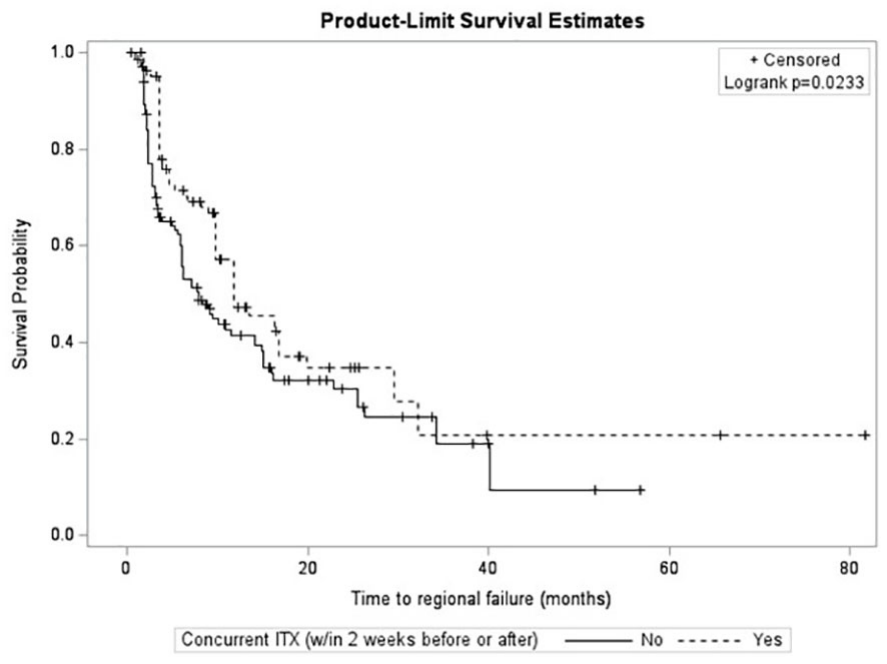
Kaplan-Meier curves for time to regional failure stratified by immunotherapy.

After survival analysis and construction of Kaplan-Meier product limit curves, metastases treated with concurrent ITX demonstrated significantly better regional failure-free survival (log-rank p=0.0233) compared to metastases treated without concurrent ITX (**Figure 2**). After univariate analyses, age at diagnosis, histology, MRI maximum axial dimension, time interval between diagnostic MRI and first treatment, time interval between CT simulation and first treatment, fractionated versus single fraction SRS, total dose, GTV, PTV, max GTV dose, presence of normal brain constraint, and a history of prior WBRT were not associated with regional failure (**Table 4**). Conversely, male gender (HR 0.56, 95% CI 0.38 – 0.81, p=0.0022), Rx IDL (HR 0.94, 95% CI 0.90 – 0.99, p=0.0216), concurrent ITX (HR 0.68, 95% CI 0.48 – 0.96, p=0.0259), and a history of prior SRS for a different lesion (HR 0.65, 95% CI 0.44 – 0.96, p=0.0295) were all associated with a lower risk of regional failure. No variables were associated with an increased risk of regional failure. Following the univariate screen, gender, Rx IDL, concurrent ITX, and a history of prior SRS to another lesion were selected to be included in the multivariate model. Tumor histology was also included in the multivariate model due to the relatively low p-value for breast metastases (HR 1.48, 95% CI 0.92 – 2.39, p=0.1050) that approached p=0.1. After multivariate analyses, only male gender (HR 0.53, 95% CI 0.35 – 0.81, p=0.0034), Rx IDL (HR 0.91, 95% CI 0.86 – 0.96, p=0.0002), and previous SRS to another lesion (HR 0.54, 95% CI 0.35 – 0.81, p=0.0031) remained associated with lower risk of regional failure. In addition, NSCLC histology (HR 0.58, 95% CI 0.38 – 0.88, p=0.0096) was also associated with lower risk of regional failure compared to other histologies.

## Discussion

In this patient cohort, a relationship between an increased treatment delay after diagnostic MRI or CT simulation and incidence of radionecrosis could not be demonstrated (**Table 1**). Furthermore, a relationship between increased treatment delay after diagnostic MRI or CT simulation and local failure could also not be reliably demonstrated (**Table 3**). Increased treatment delay after diagnostic MRI and after CT simulation were also not associated with regional failure (**Table 4**). While previous research on treatment delays prior to WBRT after diagnostic CT or MRI also demonstrated a non-significant relationship with overall survival (51), limited research has been conducted to date on the relationship between treatment delays prior to SRS and local control (45), and there is no published research on the relationship between treatment delays and regional control or the incidence of radionecrosis.

**Table 4:** Univariate and multivariable analyses for time to regional failure (distant brain failure).

Parameter		UNIVARIABLE				MULTIVARIABLE			
		Hazard ratio	95% Confidence Limits for the Hazard Ratio		*p*-value	Hazard ratio	95% Confidence Limits for the Hazard Ratio		*p*-value
**Age at Diagnosis**		1.00	0.98	1.01	0.6353				
**Histology**	*Breast*	1.48	0.92	2.39	0.1050	1.26	0.76	2.09	0.3639
	*NSCLC*	0.79	0.53	1.17	0.2354	0.58	0.38	0.88	0.0096
	*Other*	*ref*				*ref*			
**Gender**	*Male vs. Female*	0.56	0.38	0.81	0.0022	0.53	0.35	0.81	0.0034
**MRI Max Axial Dimension (mm)**		0.99	0.96	1.01	0.3711				
**Dx MRI - First Tx Interval**		0.99	0.99	1.00	0.0556				
**Sim First Tx Interval**		0.99	0.97	1.02	0.6358				
**Fractions**	*>1 vs. 1*	1.50	0.94	2.39	0.0891				
**Total Dose (cGy)**		1.00	1.00	1.00	0.8972				
**Rx IDL (%)**		0.94	0.90	0.99	0.0216	0.91	0.86	0.96	0.0002
**GTV (cc)**		0.98	0.94	1.03	0.4497				
**PTV (cc)**		0.99	0.96	1.02	0.4520				
**Max GTV Dose (cGy)**		1.00	1.00	1.00	0.4826				
**Normal Brain Constraint**	*Yes vs. No*	1.10	0.78	1.56	0.5815				
**Concurrent ITX**	*Yes vs. No*	0.68	0.48	0.96	0.0259	0.82	0.58	1.17	0.2763
**Previous SRS other lesion**	*Yes vs. No*	0.65	0.44	0.96	0.0295	0.54	0.35	0.81	0.0031
**Previous WBRT**	*Yes vs. No*	0.68	0.38	1.20	0.1821				

Seymour et al. found that a time interval longer than or equal to 14 days in between treatment planning MRI and SRS was associated with a shorter time period before local failure compared to a time interval less than 14 days in between treatment planning MRI and SRS (45). However, given the median time in between MRI and SRS in Seymour et al. was 11 days with an interquartile range of 6 to 23 days compared to the median time in between MRI and SRS in this patient cohort of 34 days with an interquartile range of 28 to 48.5 days (**Table 1**), it is possible that the association between local failure and increase in treatment delay is less significant when treatment delay increases beyond a certain point. It is also possible that the inclusion of margins during the treatment planning of this cohort compared to the relative lack of margins in 94.04% of metastases in Seymour et al. may have accounted for most tumor growth during the time interval between MRI and SRS in this cohort, resulting in the lack of association between treatment delay and local failure. In fact, another study found a 2mm margin was sufficient to cover 100% of tumor growth for 78% of metastases after a mean of 23 days in between consecutive MRI scans (48). In this cohort, an advantage to a shorter interval between diagnosis of brain metastases and treatment that minimizes radionecrosis and maximizes local and regional control was not identified. Regardless, delays in starting radiotherapy should be minimized whenever possible.

In this study, MRI maximum axial dimension and a history of prior WBRT (**Table 2**) predicted radionecrosis, both of which are well-established risk factors (11, 22–25, 32). An increase in GTV was also associated with local failure on multivariate analyses (**Table 3**), also concordant with the published literature (52, 53). While differences in histology were not associated with radionecrosis in this patient population (**Table 1**), NSCLC was associated with a lower risk of local failure compared to other histologies after multivariate analyses (**Table 3**). While this is also reflected in other studies (54, 55), contradictory reports suggest that breast cancer may be more radiosensitive (56). Unfortunately, details of histology subtype (e.g. triple negative breast cancer, squamous cell carcinoma of the lung) were not readily available that may shed additional light on these findings. Additionally, NSCLC was also associated with a lower risk of regional failure (**Table 4**) compared to other histologies after multivariate analyses, with one study suggesting a lower risk of regional failure for NSCLC compared to SCLC (55), and another study suggesting a relationship between melanoma histology and regional failure with NSCLC demonstrating a nonsignificant relationship with regional failure (57).

Male gender was associated with greater risk of local failure on multivariate analyses (**Table 3**), a finding also supported by numerous other studies (36, 58, 59), although the biological mechanism for this finding is not understood. A lower risk of regional failure was also identified with increasing Rx IDL and previous SRS to another lesion, findings that are neither intuitive nor observed in other studies to the best of the authors’ knowledge (**Table 4**). While one might suspect that prior off-target failure would predict additional sites of intracranial failure following SRS, it is possible that such patients have more advanced disease and died before regional failure was established. Contrarily, those patients undergoing initial radiosurgery are likely earlier in their disease course and have a higher risk of regional failure due to a longer life expectancy.

Interestingly, while concurrent ITX was not associated with radionecrosis (**Table 1**), it was associated with better local control (**Table 3**). Metastases treated with concurrent ITX also demonstrated significantly better local failure-free survival compared to metastases treated without concurrent ITX (**Figure 1**). These findings add to the growing body of literature suggesting that the use of concurrent immunotherapies with SRS is not only safe, but enhances the efficacy of radiosurgery (34–37), though much of the existing literature is in the context of melanoma brain metastases (38–43). A potential mechanism for the synergistic effect of combining concurrent immunotherapy and SRS is the abscopal effect, in which tumor neoantigens are generated by radiation and are subsequently absorbed by antigen-presenting cells (APCs) that then activate CD8^+^ T cells (60–63). While the immune response can typically be modulated by proteins such as cytotoxic T lymphocyte-associated antigen 4 (CTLA-4), programmed cell death 1 (PD-1), and programmed death-ligand 1 (PD-L1), immunotherapies inhibit these proteins and act as immune checkpoint inhibitors, thereby increasing immune system activation (60, 61).

Limitations of this study include the retrospective nature and exclusion of some patients due to lack of follow-up imaging. Histopathologic confirmation of radionecrosis was also not available for all metastases. Furthermore, concurrent immunotherapies were not separated based upon the targets of the inhibitors and analysis was not separated based upon tumor histology. Other systemic therapies may have also been utilized during treatment that were not recorded or analyzed. Nonetheless, this study is the first to the authors’ knowledge assessing the impact of treatment delays on radionecrosis and regional failure, and this study adds to the limited published data on the relationship between treatment delays and local failure.

## Conclusion

In this patient cohort, there was no relationship between treatment interval from either diagnostic MRI or CT simulation to treatment and incidence of radionecrosis, local failure, and regional failure; consequently, an optimal interval between diagnosis of intact brain metastases and radiosurgery could not be identified. Concurrent ITX was not associated with radionecrosis, but was associated with a lower risk of local failure, suggesting a synergistic oncologic effect without an attendant increase in toxicity.

## Data availability statement

The raw data supporting the conclusions of this article will be made available by the authors, without undue reservation.

## Ethics statement

This study involved human participants and was reviewed and approved by the NYU Langone Long Island Institutional Review Board. Written informed consent for participation was not required for this study in accordance with the national legislation and the institutional requirements.

## Author contributions

TC, PD, LT, and MCR conceived the project. JL, CM, and MCR collected the data and constructed the dataset. Statistical analysis was performed by MA. JL, MA, and MCR wrote an initial draft of the manuscript. All authors had approval over the final copy of the manuscript. All authors contributed to the article and approved the submitted version.

## References

[1] Johnson JD Young B. Demographics of brain metastasis. Neurosurg Clin N Am (1996) 7(3):337–44. doi: 10.1016/S1042-3680(18)30365-6 8823767

[2] Nayak L Lee EQ Wen PY. Epidemiology of brain metastases. Curr Oncol Rep (2012) 14(1):48–54. doi: 10.1007/s11912-011-0203-y 22012633

[3] Lin X DeAngelis LM. Treatment of brain metastases. J Clin Oncol (2015) 33(30):3475–84. doi: 10.1200/JCO.2015.60.9503 PMC5087313 26282648

[4] Brown PD Ahluwalia MS Khan OH Asher AL Wefel JS Gondi V. Whole-brain radiotherapy for brain metastases: Evolution or revolution? J Clin Oncol (2018) 36(5):483–91. doi: 10.1200/JCO.2017.75.9589 PMC6075843 29272161

[5] Leksell L. The stereotaxic method and radiosurgery of the brain. Acta Chir Scand (1951) 102(4):316–9. 14914373

[6] Shinde A Akhavan D Sedrak M Glaser S Amini A. Shifting paradigms: whole brain radiation therapy versus stereotactic radiosurgery for brain metastases. CNS Oncol (2019) 8(1):CNS27. doi: 10.2217/cns-2018-0016 30701987 PMC6499015

[7] Vogelbaum MA Brown PD Messersmith H Brastianos PK Burri S Cahill D. Treatment for brain metastases: ASCO-SNO-ASTRO guideline. J Clin Oncol (2022) 40(5):492–516. doi: 10.1200/JCO.21.02314 34932393

[8] Lippitz B Lindquist C Paddick I Peterson D O’Neill K Beaney R. Stereotactic radiosurgery in the treatment of brain metastases: The current evidence. Cancer Treat Rev (2014) 40(1):48–59. doi: 10.1016/j.ctrv.2013.05.002 23810288

[9] Aoyama H Shirato H Tago M Nakagawa K Toyoda T Hatano K. Stereotactic radiosurgery plus whole-brain radiation therapy vs stereotactic radiosurgery alone for treatment of brain metastases: A randomized controlled trial. JAMA (2006) 295(21):2483–91. doi: 10.1001/jama.295.21.2483 16757720

[10] Brown PD Jaeckle K Ballman KV Farace E Cerhan JH Anderson SK. Effect of radiosurgery alone vs radiosurgery with whole brain radiation therapy on cognitive function in patients with 1 to 3 brain metastases: A randomized clinical trial. JAMA (2016) 316(4):401–9. doi: 10.1001/jama.2016.9839 PMC5313044 27458945

[11] Le Rhun E Dhermain F Vogin G Reyns N Metellus P. Radionecrosis after stereotactic radiotherapy for brain metastases. Expert Rev Neurother (2016) 16(8):903–14. doi: 10.1080/14737175.2016.1184572 27177183

[12] Kayama T Sato S Sakurada K Mizusawa J Nishikawa R Narita Y. Effects of surgery with salvage stereotactic radiosurgery versus surgery with whole-brain radiation therapy in patients with one to four brain metastases (JCOG0504): A phase III, noninferiority, randomized controlled trial. J Clin Oncol (2018) 36(33):3282–9. doi: 10.1200/JCO.2018.78.6186 29924704

[13] Vellayappan B Tan CL Yong C Khor LK Koh WY Yeo TT. Diagnosis and management of radiation necrosis in patients with brain metastases. Front Oncol (2018) 8:395. doi: 10.3389/fonc.2018.00395 30324090 PMC6172328

[14] Strenger V Lackner H Mayer R Sminia P Sovinz P Mokry M. Incidence and clinical course of radionecrosis in children with brain tumors. a 20-year longitudinal observational study. Strahlenther Onkol Organ Dtsch Rontgengesellschaft Al (2013) 189(9):759–64. doi: 10.1007/s00066-013-0408-0 23963155

[15] Remler MP Marcussen WH Tiller-Borsich J. The late effects of radiation on the blood brain barrier. Int J Radiat Oncol Biol Phys (1986) 12(11):1965–9. doi: 10.1016/0360-3016(86)90133-1 3771316

[16] Panagiotakos G Alshamy G Chan B Abrams R Greenberg E Saxena A. Long-term impact of radiation on the stem cell and oligodendrocyte precursors in the brain. PloS One (2007) 2(7):e588. doi: 10.1371/journal.pone.0000588 17622341 PMC1913551

[17] Chao ST Ahluwalia MS Barnett GH Stevens GHJ Murphy ES Stockham AL. Challenges with the diagnosis and treatment of cerebral radiation necrosis. Int J Radiat Oncol (2013) 87(3):449–57. doi: 10.1016/j.ijrobp.2013.05.015 23790775

[18] Ali FS Arevalo O Zorofchian S Patrizz A Riascos R Tandon N. Cerebral radiation necrosis: Incidence, pathogenesis, diagnostic challenges, and future opportunities. Curr Oncol Rep (2019) 21(8):66. doi: 10.1007/s11912-019-0818-y 31218455

[19] Flickinger JC Kondziolka D Lunsford LD Kassam A Phuong LK Liscak R. Development of a model to predict permanent symptomatic postradiosurgery injury for arteriovenous malformation patients. arteriovenous malformation radiosurgery study group. Int J Radiat Oncol Biol Phys (2000) 46(5):1143–8. doi: 10.1016/S0360-3016(99)00513-1 10725624

[20] Ohtakara K Hayashi S Nakayama N Ohe N Yano H Iwama T. Significance of target location relative to the depth from the brain surface and high-dose irradiated volume in the development of brain radionecrosis after micromultileaf collimator-based stereotactic radiosurgery for brain metastases. J Neurooncol (2012) 108(1):201–9. doi: 10.1007/s11060-012-0834-3 22392126

[21] Miller JA Bennett EE Xiao R Kotecha R Chao ST Vogelbaum MA. Association between radiation necrosis and tumor biology after stereotactic radiosurgery for brain metastasis. Int J Radiat Oncol Biol Phys (2016) 96(5):1060–9. doi: 10.1016/j.ijrobp.2016.08.039 27742540

[22] Brown PD Ballman KV Cerhan JH Anderson SK Carrero XW Whitton AC. Postoperative stereotactic radiosurgery compared with whole brain radiotherapy for resected metastatic brain disease (NCCTG N107C/CEC·3): a multicentre, randomised, controlled, phase 3 trial. Lancet Oncol (2017) 18(8):1049–60. doi: 10.1016/S1470-2045(17)30441-2 PMC5568757 28687377

[23] Shaw E Scott C Souhami L Dinapoli R Bahary JP Kline R. Radiosurgery for the treatment of previously irradiated recurrent primary brain tumors and brain metastases: Initial report of radiation therapy oncology group protocol (90-05). Int J Radiat Oncol Biol Phys (1996) 34(3):647–54. doi: 10.1016/0360-3016(95)02106-X 8621289

[24] Shaw E Scott C Souhami L Dinapoli R Kline R Loeffler J. Single dose radiosurgical treatment of recurrent previously irradiated primary brain tumors and brain metastases: final report of RTOG protocol 90-05. Int J Radiat Oncol Biol Phys (2000) 47(2):291–8. doi: 10.1016/S0360-3016(99)00507-6 10802351

[25] Mahajan A Ahmed S McAleer MF Weinberg JS Li J Brown P. Post-operative stereotactic radiosurgery versus observation for completely resected brain metastases: A single-centre, randomised, controlled, phase 3 trial. Lancet Oncol (2017) 18(8):1040–8. doi: 10.1016/S1470-2045(17)30414-X PMC5560102 28687375

[26] Blonigen BJ Steinmetz RD Levin L Lamba MA Warnick RE Breneman JC. Irradiated volume as a predictor of brain radionecrosis after linear accelerator stereotactic radiosurgery. Int J Radiat Oncol Biol Phys (2010) 77(4):996–1001. doi: 10.1016/j.ijrobp.2009.06.006 19783374

[27] Ruben JD Dally M Bailey M Smith R McLean CA Fedele P. Cerebral radiation necrosis: Incidence, outcomes, and risk factors with emphasis on radiation parameters and chemotherapy. Int J Radiat Oncol (2006) 65(2):499–508. doi: 10.1016/j.ijrobp.2005.12.002 16517093

[28] Rahmathulla G Marko NF Weil RJ. Cerebral radiation necrosis: A review of the pathobiology, diagnosis and management considerations. J Clin Neurosci (2013) 20(4):485–502. doi: 10.1016/j.jocn.2012.09.011 23416129

[29] Lehrer EJ Peterson JL Zaorsky NG Brown PD Sahgal A Chiang VL. Single versus multifraction stereotactic radiosurgery for Large brain metastases: An international meta-analysis of 24 trials. Int J Radiat Oncol Biol Phys (2019) 103(3):618–30. doi: 10.1016/j.ijrobp.2018.10.038 30395902

[30] Minniti G Scaringi C Paolini S Lanzetta G Romano A Cicone F. Single-fraction versus multifraction (3 × 9 gy) stereotactic radiosurgery for Large (>2 cm) brain metastases: A comparative analysis of local control and risk of radiation-induced brain necrosis. Int J Radiat Oncol Biol Phys (2016) 95(4):1142–8. doi: 10.1016/j.ijrobp.2016.03.013 27209508

[31] Wegner RE Leeman JE Kabolizadeh P Rwigema JC Mintz AH Burton SA. Fractionated stereotactic radiosurgery for large brain metastases. Am J Clin Oncol (2015) 38(2):135–9. doi: 10.1097/COC.0b013e31828aadac 23563213

[32] Sneed PK Mendez J Vemer-van den Hoek JGM Seymour ZA Ma L Molinaro AM. Adverse radiation effect after stereotactic radiosurgery for brain metastases: Incidence, time course, and risk factors. J Neurosurg (2015) 123(2):373–86. doi: 10.3171/2014.10.JNS141610 25978710

[33] Kocher M Wittig A Piroth MD Treuer H Seegenschmiedt H Ruge M. Stereotactic radiosurgery for treatment of brain metastases. a report of the DEGRO working group on stereotactic radiotherapy. Strahlenther Onkol Organ Dtsch Rontgengesellschaft Al (2014) 190(6):521–32. doi: 10.1007/s00066-014-0648-7 24715242

[34] Chen L Douglass J Kleinberg L Ye X Marciscano AE Forde PM. Concurrent immune checkpoint inhibitors and stereotactic radiosurgery for brain metastases in non-small cell lung cancer, melanoma, and renal cell carcinoma. Int J Radiat Oncol Biol Phys (2018) 100(4):916–25. doi: 10.1016/j.ijrobp.2017.11.041 29485071

[35] Lanier CM Hughes R Ahmed T LeCompte M Masters AH Petty WJ. Immunotherapy is associated with improved survival and decreased neurologic death after SRS for brain metastases from lung and melanoma primaries. Neuro-Oncol Pract (2019) 6(5):402–9. doi: 10.1093/nop/npz004 PMC6753360 31555455

[36] Asher AL Alvi MA Bydon M Pouratian N Warnick RE McInerney J. Local failure after stereotactic radiosurgery (SRS) for intracranial metastasis: analysis from a cooperative, prospective national registry. J Neurooncol (2021) 152(2):299–311. doi: 10.1007/s11060-021-03698-7 33481148

[37] Hubbeling HG Schapira EF Horick NK Goodwin KEH Lin JJ Oh KS. Safety of combined PD-1 pathway inhibition and intracranial radiation therapy in non-small cell lung cancer. J Thorac Oncol Off Publ Int Assoc Study Lung Cancer (2018) 13(4):550–8. doi: 10.1016/j.jtho.2018.01.012 29378267

[38] Kiess AP Wolchok JD Barker CA Postow MA Tabar V Huse JT. Stereotactic radiosurgery for melanoma brain metastases in patients receiving ipilimumab: Safety profile and efficacy of combined treatment. Int J Radiat Oncol Biol Phys (2015) 92(2):368–75. doi: 10.1016/j.ijrobp.2015.01.004 PMC4955924 25754629

[39] Gabani P Fischer-Valuck BW Johanns TM Hernandez-Aya LF Keller JW Rich KM. Stereotactic radiosurgery and immunotherapy in melanoma brain metastases: Patterns of care and treatment outcomes. Radiother Oncol J Eur Soc Ther Radiol Oncol (2018) 128(2):266–73. doi: 10.1016/j.radonc.2018.06.017 29960685

[40] Knisely JPS Yu JB Flanigan J Sznol M Kluger HM Chiang VLS. Radiosurgery for melanoma brain metastases in the ipilimumab era and the possibility of longer survival. J Neurosurg (2012) 117(2):227–33. doi: 10.3171/2012.5.JNS111929 PMC6098938 22702482

[41] Silk AW Bassetti MF West BT Tsien CI Lao CD. Ipilimumab and radiation therapy for melanoma brain metastases. Cancer Med (2013) 2(6):899–906. doi: 10.1002/cam4.140 24403263 PMC3892394

[42] Acharya S Mahmood M Mullen D Yang D Tsien CI Huang J. Distant intracranial failure in melanoma brain metastases treated with stereotactic radiosurgery in the era of immunotherapy and targeted agents. Adv Radiat Oncol (2017) 2(4):572–80. doi: 10.1016/j.adro.2017.07.003 PMC5707419 29204524

[43] Choong ES Lo S Drummond M Fogarty GB Menzies AM Guminski A. Survival of patients with melanoma brain metastasis treated with stereotactic radiosurgery and active systemic drug therapies. Eur J Cancer (2017) 75:169–78. doi: 10.1016/j.ejca.2017.01.007 28236768

[44] Colaco RJ Martin P Kluger HM Yu JB Chiang VL. Does immunotherapy increase the rate of radiation necrosis after radiosurgical treatment of brain metastases? J Neurosurg (2016) 125(1):17–23. doi: 10.3171/2015.6.JNS142763 26544782

[45] Seymour ZA Fogh SE Westcott SK Braunstein S Larson DA Barani IJ. Interval from imaging to treatment delivery in the radiation surgery age: How long is too long? Int J Radiat Oncol Biol Phys (2015) 93(1):126–32. doi: 10.1016/j.ijrobp.2015.05.001 26279030

[46] Yoo H Jung E Nam BH Shin SH Gwak HS Kim MS. Growth rate of newly developed metastatic brain tumors after thoracotomy in patients with non-small cell lung cancer. Lung Cancer Amst Neth (2011) 71(2):205–8. doi: 10.1016/j.lungcan.2010.05.013 20570390

[47] Kobets AJ Backus R Fluss R Lee A Lasala PA. Evaluating the natural growth rate of metastatic cancer to the brain. Surg Neurol Int (2020) 11:254. doi: 10.25259/SNI_291_2020 33024592 PMC7533080

[48] Garcia MA Anwar M Yu Y Duriseti S Merritt B Nakamura J. Brain metastasis growth on preradiosurgical magnetic resonance imaging. Pract Radiat Oncol (2018) 8(6):e369–76. doi: 10.1016/j.prro.2018.06.004 30174247

[49] Zeger SL Liang KY. Longitudinal data analysis for discrete and continuous outcomes. Biometrics (1986) 42(1):121–30. doi: 10.2307/2531248 3719049

[50] Zeger SL Liang KY Albert PS. Models for longitudinal data: A generalized estimating equation approach. Biometrics (1988) 44(4):1049–60. doi: 10.2307/2531734 3233245

[51] Hansen HC Janssen S Thieme C Perlov A Schild SE Rades D. Whole-brain radiotherapy (WBRT) for brain metastases: Does the interval between imaging and treatment matter? Anticancer Res (2018) 38(12):6835–40. doi: 10.21873/anticanres.13057 30504398

[52] Ogura K Mizowaki T Ogura M Sakanaka K Arakawa Y Miyamoto S. Outcomes of hypofractionated stereotactic radiotherapy for metastatic brain tumors with high risk factors. J Neurooncol (2012) 109(2):425–32. doi: 10.1007/s11060-012-0912-6 22714054

[53] Baschnagel AM Meyer KD Chen PY Krauss DJ Olson RE Pieper DR. Tumor volume as a predictor of survival and local control in patients with brain metastases treated with gamma knife surgery. J Neurosurg (2013) 119(5):1139–44. doi: 10.3171/2013.7.JNS13431 23971958

[54] Woo HJ Hwang SK Park SH Hwang JH Hamm IS. Factors related to the local treatment failure of γ knife surgery for metastatic brain tumors. Acta Neurochir (Wien) (2010) 152(11):1909–14. doi: 10.1007/s00701-010-0805-4 20890616

[55] Kuremsky JG Urbanic JJ Petty WJ Lovato JF Bourland JD Tatter SB. Tumor histology predicts patterns of failure and survival in patients with brain metastases from lung cancer treated with gamma knife radiosurgery. Neurosurgery (2013) 73(4):641–7. doi: 10.1227/NEU.0000000000000072 PMC3880778 23842552

[56] Black PJ Page BR Lucas JT Hughes RT Laxton AW Tatter SB. Factors that determine local control with gamma knife radiosurgery: The role of primary histology. J Radiosurgery SBRT (2015) 3(4):281–6. PMC4605606 26478823

[57] Sawrie SM Guthrie BL Spencer SA Nordal RA Meredith RF Markert JM. Predictors of distant brain recurrence for patients with newly diagnosed brain metastases treated with stereotactic radiosurgery alone. Int J Radiat Oncol (2008) 70(1):181–6. doi: 10.1016/j.ijrobp.2007.05.084 17768015

[58] Molenaar R Wiggenraad R Verbeek-de Kanter A Walchenbach R Vecht C. Relationship between volume, dose and local control in stereotactic radiosurgery of brain metastasis. Br J Neurosurg (2009) 23(2):170–8. doi: 10.1080/02688690902755613 19306173

[59] Mangesius J Seppi T Bates K Arnold CR Minasch D Mangesius S. Hypofractionated and single-fraction radiosurgery for brain metastases with sex as a key predictor of overall survival. Sci Rep (2021) 11(1):8639. doi: 10.1038/s41598-021-88070-5 33883632 PMC8060341

[60] Ngwa W Irabor OC Schoenfeld JD Hesser J Demaria S Formenti SC. Using immunotherapy to boost the abscopal effect. Nat Rev Cancer (2018) 18(5):313–22. doi: 10.1038/nrc.2018.6 PMC5912991 29449659

[61] Liu Y Dong Y Kong L Shi F Zhu H Yu J. Abscopal effect of radiotherapy combined with immune checkpoint inhibitors. J Hematol OncolJ Hematol Oncol (2018) 11:104. doi: 10.1186/s13045-018-0647-8 30115069 PMC6097415

[62] Chen L Douglass J Walker AJ Marciscano AE Lim M Kleinberg LR. Concurrent immunotherapy and stereotactic radiosurgery for brain metastases is associated with a decreased incidence of new intracranial metastases. Int J Radiat Oncol Biol Phys (2015) 93(3):E102. doi: 10.1016/j.ijrobp.2015.07.807

[63] Singh SA McDermott DM Mattes MD. Impact of systemic therapy type and timing on intracranial tumor control in patients with brain metastasis from non-Small-Cell lung cancer treated with stereotactic radiosurgery. World Neurosurg (2020) 144:e813–23. doi: 10.1016/j.wneu.2020.09.082 32956881

