# Corrigendum: Time interval from diagnosis to treatment of brain metastases with stereotactic radiosurgery is not associated with radionecrosis or local failure

**DOI:** 10.3389/fonc.2023.1192726

**Published:** 2023-04-05

**Authors:** Justin Leu, Meredith Akerman, Christopher Mendez, Jonathan W. Lischalk, Todd Carpenter, David Ebling, Jonathan A. Haas, Matthew Witten, Marissa Barbaro, Paul Duic, Lee Tessler, Michael C. Repka

**Affiliations:** ^1^ Renaissance School of Medicine, Stony Brook University, Stony Brook, NY, United States; ^2^ Division of Health Services Research, New York University (NYU) Long Island School of Medicine, Mineola, NY, United States; ^3^ Department of Radiation Oncology, Perlmutter Cancer Center at New York University (NYU) Long Island, Mineola, NY, United States; ^4^ NYCyberKnife at Perlmutter Cancer Center – Manhattan, New York, NY, United States; ^5^ Department of Medical Physics, Perlmutter Cancer Center at New York University (NYU) Long Island, Mineola, NY, United States; ^6^ Department of Neurology, New York University (NYU) Long Island School of Medicine, Mineola, NY, United States; ^7^ Department of Neurosurgery, Perlmutter Cancer Center at New York University (NYU) Long Island, Mineola, NY, United States; ^8^ Department of Radiation Oncology, University of North Carolina School of Medicine, Chapel Hill, NC, United States

**Keywords:** stereotactic radiosurgery, brain metastases, immunotherapy, radionecrosis, treatment delays, cancer, radiation therapy, radiation necrosis

**Text Correction**

In the published article, there was an error. One of the citations of Table 3 was incorrect and should have referred to Table 4 instead.

A correction has been made to **the Results section**, *Association between regional failure (distant brain failure) and other variables*, paragraph 1. This sentence previously stated:

“After univariate analyses, age at diagnosis, histology, MRI maximum axial dimension, time interval between diagnostic MRI and first treatment, time interval between CT simulation and first treatment, fractionated versus single fraction SRS, total dose, GTV, PTV, max GTV dose, presence of normal brain constraint, and a history of prior WBRT were not associated with regional failure (Table 3).”

The corrected sentence appears below:

“After univariate analyses, age at diagnosis, histology, MRI maximum axial dimension, time interval between diagnostic MRI and first treatment, time interval between CT simulation and first treatment, fractionated versus single fraction SRS, total dose, GTV, PTV, max GTV dose, presence of normal brain constraint, and a history of prior WBRT were not associated with regional failure (Table 4).”

The authors apologize for this error and state that this does not change the scientific conclusions of the article in any way. The original article has been updated.

